# Ecotype Variation in Trace Element Content of Hard Tissues in the European Roe Deer (*Capreolus capreolus*)

**DOI:** 10.1007/s00244-018-0580-4

**Published:** 2018-11-15

**Authors:** Jan Demesko, Janusz Markowski, Eva Demesko, Mirosława Słaba, Janusz Hejduk, Piotr Minias

**Affiliations:** 1 Department of Biodiversity Studies and Bioeducation, Faculty of Biology and Environmental Protection, University of Łódź, Banacha 1/3, 90-237 Lodz, Poland; 2 Faculty of Medicine with Dentistry Division, Medical University of Lublin, Al. Racławickie 1, 20-059 Lublin, Poland; 3 Department of Industrial Microbiology and Biotechnology, Faculty of Biology and Environmental Protection, University of Łódź, Banacha 12/16, 90-237 Lodz, Poland

## Abstract

Animals living in anthropogenic habitats bear a multitude of costs, which are directly or indirectly associated with human activities. Among others, an elevated exposure to environmental pollution can have negative consequences for wildlife populations. We examined the differences in the concentrations of trace elements between the field and forest ecotype of the European roe deer (*Capreolus capreolus*). Naturally, roe deer inhabited various types of woodlands (forest ecotype), but within the last century, they adapted to life in a human-transformed agricultural areas (field ecotype), which could be associated with an increased exposure to pollution. In this study, we measured concentrations of seven trace metals (barium, copper, iron, lead, manganese, strontium, zinc) and fluoride in skull bones and permanent teeth of more than 230 roe deer from 8 study plots in East-Central Europe. We found that field roe deer had higher concentrations of four trace metals (copper, iron, lead, strontium) and fluoride compared with forest roe deer. These differences were consistent with variations in the general level of environmental contamination within the study plots, as assessed with trace element content in wild plants. Our study indicates that bone and teeth of the European roe deer can be used as a valid indicator of environmental pollution. Also, we expect that elevated exposure of field roe deer to environmental pollution can have negative consequences for wild populations of this species, as well as for the consumers of venison.

**Electronic supplementary material:**

The online version of this article (10.1007/s00244-018-0580-4) contains supplementary material, which is available to authorized users.

Rapidly increasing urbanization and industrialization in the second half of XX and the first decade of XXI century have led to large amounts of toxic contaminants being released into the environment worldwide. Many toxic elements occur naturally in the environment, but their concentrations can increase dramatically as a result of anthropogenic activities, such as mining, metal smelting, coal-based energy production, solid waste incineration, industrial manufacturing, as well as erosion of road surfaces by traffic and the abrasion of brakes and tires (Nriagu 1996; WHO 2013; Clemens and Ma 2016). Modern agricultural practices also contribute to increasing environmental pollution via application of agrochemicals and inorganic fertilizers (Chauhan et al. 2012). As a result, environmental concentrations of toxic elements can substantially exceed their normal background level, which disturbs biological balance of ecosystems and produces harmful effects on wildlife and human health (Tchounwou et al. 2012; Jaishankar et al. 2014), requiring implementation of pollution monitoring procedures (Wolkers et al. 1994; Srebočan et al. 2011). Wild animals, especially game species, are used relatively often as bioindicators of environmental pollution. In Europe, extensive ecotoxicological research has been conducted on cervids, which usually have wide geographical distribution and relatively long life-span (Sawicka-Kapusta 1979; Frank 1986; Tataruch and Kierdorf 2003). Also, their meat is a valuable and desired component of the human diet (Jarzyńska and Falandysz 2011), which has to comply with the World Health Organization standards for the content of heavy metal as pollutants (Lehel et al. 2016).

The European roe deer (*Capreolus capreolus*) is recognized as one of the most ecologically plastic species among cervids, because it can tolerate strong anthropogenic pressure and can thrive in a human-impacted landscape (Augustine and McNaughton 1998; Tinoco Torres et al. 2011). Although the natural habitats of roe deer include a wide variety of forest types, the species has adapted to life in an intensively cultivated agricultural land across large areas of Europe (Pielowski 1984; Ellenberg 1978). Based on divergence in habitat selection, field and forest dwelling roe deer were identified as different ecotypes (Pielowski 1984). These two ecotypes have been reported to show remarkable phenotypic differences in morphology and anatomy (Fruziński et al. 1982; Hofmann et al. 1988; Pėtelis and Brazaitis 2003; Flis 2011), ecology (Zejda and Homolka 1980, Pielowski and Bresiński 1982; Kałuziński 1982; Zejda and Bauerova 1985), and physiology (Majewska et al. 1982). Jeppesen (1990) estimated that home range of forest ecotype varies from 15 to 85 ha, while it was at least twice bigger in the field ecotype. Forest and field ecotypes of roe deer also differ in feeding preferences (Tixier and Duncan 1996). The diet of the field roe deer is primarily based on cultivated plants, which may constitute up to 66% of the total feed mass (Kałuziński 1982). In contrast, forest deer ecotype typically feeds on the shoots of shrubs and trees, as well as wild herbaceous grasses and plants (Gębczynska 1980).

Heavy metal content in wildlife can be assessed across different types of tissues, which substantially vary in an average turnover time of their elements. For example, analysis of body fluids (e.g., serum, urine, or cerebrospinal fluid), which have the highest turnover rate, is only useful for evaluation of short-term exposure to pollutants, and thus, these tissues are rarely used in ecotoxicological research (Baroni et al. 2000; Humann-Ziehank et al. 2008; Žele and Vengušt 2012). In contrast, analysis of soft tissues and internal organs, especially liver and kidney, which accumulate toxic elements, can capture longer periods of exposure to contamination and these types of tissues have commonly been used in ecotoxicological monitoring of roe deer (Kryński et al. 1982; Frank 1986; Babińska-Werka and Czarnowska 1988; Pokorny and Ribarič-Lasnik 2002; Pompe-Gotal and Prevendar-Crnić 2002; de Mendoza et al. 2011; Srebočan et al. 2011; Długaszek and Kopczyński 2013; Wieczorek-Dabrowska et al. 2013; Durkalec et al. 2015; Lehel et al. 2016). Finally, hard tissues, such as bone or teeth, have the lowest turnover rates, and they are known to accumulate trace elements over years or decades (Glimcher 2006). For example, the biological half-life of trace elements in human bone tissue is up to 30 years, and the content of heavy metals in bones is known to comprise up to 90% of their total body content (Zaichick et al. 2011). In cervids, heavy metal content has been commonly assessed in antlers, because they are regularly collected as hunting trophies and can easily be used as research material (Kierdorf and Kierdorf 2002, 2004, 2006). However, cervids usually produce new antlers each year, and thus, they are not particularly suitable to investigate long-term exposure to pollutants. Taking all this into account, bone and teeth are expected to more reliably indicate long-term bioaccumulation of pollutants, and consistent with this prediction, we have recently shown that heavy metal content of permanent teeth reliably indicate throughout-life intoxication by environmental pollution in the European roe deer (Demesko et al. 2018).

The purpose of this study was to test for the differences in trace element content between the field and the forest ecotype of the Eurasian roe deer. For this purpose, we measured concentrations of seven trace metals (barium, copper, iron, lead, manganese, strontium, zinc) and fluoride in skull bones and permanent teeth of more than 230 roe deer from 8 study plots in East-Central Europe. We predicted higher trace element concentrations in the field ecotype of roe deer, which could be due to: (1) differences in general environmental pollution between areas inhabited by the two ecotypes of roe deer, or (2) difference in the ecology of the two roe deer ecotypes, i.e., an alteration in diet composition from wild forest plants (forest ecotype) to cultivated crops (field ecotype). To estimate the level of general environmental pollution within each study plot, we measured trace metal content in the common forest plants (2 species of trees and 2 genera of wild fruit plants) that are an important component of roe deer diet.

## Materials and Methods

### Study Area and Classification of Ecotypes

Samples were collected in seven game breeding centres from Łódź voivodship, Central Poland: Brzeziny (51°45′N, 19°43′E; *n* = 25), Kolumna (51°34′N, 19°13′E; *n* = 11), Kutno (52°14′N, 19°08′E; *n* = 26), Poddębice (51°54′N, 18°53′E; *n* = 11), Smardzewice (51°26′N, 19°60′E; *n* = 32), Spała (51°31′N, 20°11′E; *n* = 13), Wieluń (51°11′N, 18°44′E; *n* = 21), and in one game breeding centre from Vilnius area, Lithuania: Mickunai forest (54°41′N, 25°35′E; *n* = 94). Polish game breeding centres were located relatively close (25–100 km) to a large urban centre, Łódź (51°46′N, 19°28′E; 293 km^2^, 708 500 inhabitants), while Mickunai forest was located ca. 20 km from the Vilnius city (54°41′N, 25°17′E; 401 km^2^, 574,200 inhabitants). The share of urbanized areas within the study plots ranged from 5.2 to 26.8% (mean 9.0 ± 2.6%). The distinction between the field and forest roe deer ecotype was based on the share of woodland area within the study plots. Forest ecotype was defined as inhabiting areas with > 50% share of woodland, while field ecotype was identified in the study plots with < 35% share of woodlands (there were no study plots with 35–50% share of woodlands). Determination of roe deer ecotypes was consistent with legal classification of forest and field hunting units (Flis 2011) and with morphological variation of roe deer within our dataset, showing that forest individuals were significantly smaller than field individuals (as measured with height at the withers and chest circumference; *P* < 0.05). Mean share of woodland and agricultural areas was 58.2 ± 4.6% versus 36.1 ± 4.7% for the forest ecotype, and 22.7 ± 4.8% versus 66.2 ± 5.3% for the field ecotype. In total, samples for the field ecotype were collected in five study plots (Brzeziny, Kolumna, Kutno, Poddębice, and Wieluń), while forest ecotype was sampled in three study plots (Mickunai, Smardzewice, and Spała). There were no significant differences in the level of environmental contamination, as measured with concentrations of six heavy metals (barium, copper, iron, lead, strontium, and zinc) in wild forest plant species (see details below) between Polish and Lithuanian forest study plots (all *P* > 0.05), which provided support for our joint analysis of these data. The only difference was found for the manganese concentration in wild forest plants, which was higher in Polish than Lithuanian forest study plots (*P* < 0.001).

### Sample Collection

Roe deer were culled during regular hunting period and in accordance with local hunting plans and regulations during 2009–2015. A total sample of 233 skulls was collected (139 and 94 samples for the forest and field ecotype, respectively). Age of sampled specimens varied between 2 and 12 years, as assessed based on dental wear by the members of the Regional Commissions for Hunting Evaluation (details in Demesko et al. 2018). For the purpose of analyses, four age classes were recognized: (i) 2 years old (*n* = 77), (ii) 3–4 years old (*n* = 70), (iii) 5–6 years old (*n* = 49), and (iv) > 6 years old (*n* = 27). A small part (ca. 0.7 g of dry mass) of mandible located between foramen mental and front edge of premolar, as well as the left third permanent molar were collected from each animal using a diamond saw. Material from the entire teeth was included in the analysis, because there may be differences in the mineral composition between crown and root, as well as between dentine and enamel (Vieira et al. 2004, 2005).

To assess the level of environmental pollution within the study plots, we also collected samples of four wild forest plant species. A total of 96 plant samples were collected in the corresponding 8 game breeding areas during June 2015. Plant specimens collected included silver birch (*Betula pendula*)—leaves, Scots pine (*Pinus sylvestris*)—needles, blackberry (*Rubus ssp.*)—entire plant, and European blueberry (*Vaccinium myrtilus*)—entire plant. Samples from each plant taxon were collected from three specimens located in different parts of each study plot; however, three samples of blueberry and one sample of pine were excluded from analyses for technical reasons. Ten leaves from birch and ten needles from pine were collected at the height of up to 1 m from the ground.

### Measurements of Trace Metal and Fluoride Concentrations

All samples were washed in deionized water to remove any elements absorbed at the surface. Bone and tooth samples were powdered in a ball mill Mixer Mill MM 400 (Retsch, Germany) with zirconium oxide beads (frequency 25 Hz, time 60 s) and dried in an oven at 70 °C for 48 h. Plant samples were dried at 70 °C for 24 h. After drying, all samples were weighed to the nearest 0.01 g and 0.1 g of each sample was taken for the measurements of seven trace metal concentrations (barium, copper, iron, lead, manganese, strontium, and zinc). First, each sample was dissolved in solution of nitric acid (65%) and deionized water in the proportion of 1:15, kept in 20 °C for 24 h, and then digested at 105 °C for another 24 h using a graphite digestion block (DigiPREP Mini, SCP Science, Quebec, Canada). After digestion, all samples were diluted with deionized water to the total volume of 30 mL and stored in polypropylene metal-free vials at 20 °C until analysis.

Trace metal concentrations were measured with atomic absorption spectrophotometer SpectrAA 300A AAS, GTA-96 graphite tube atomizer, and programmable sample dispenser (Varian Techtron, Melbourne, Australia). The analyses were performed in the Laboratory of Computer and Analytic Techniques, Faculty of Biology and Environmental Protection, University of Łódź. Certified reference material from the Institute for Reference Materials and Measurements (Geel, Belgium) were used for each measurement as a quality control: ERM-186 pig kidney for copper, iron, lead, manganese, and zinc; Strontium Standard for AAS (TraceCERT^®^, 1000 mg/L Sr in nitric acid) for strontium; and Barium Standard for AAS (TraceCERT^®^, 1000 mg/L Ba in nitric acid) for barium. Recovery rates for the certified reference materials were within an acceptable margin.

Measurements of fluoride concentration followed the methodology recommended by Campus et al. (2007): 1.2 g of each bone and tooth sample was transferred to a volumetric flask, dissolved in 8 mL of 37% HCl solution, and then diluted with deionized water to the total volume of 10 mL. A 5 mL aliquot of the above solution was transferred to another volumetric flask, diluted 1:1 with deionized water, neutralized with a 6 M NaOH solution to pH 4.5, and diluted with deionized water to the total volume of 25 mL. Sample solution was diluted with TISAB (1:1), and fluoride concentration was measured with ion-selective fluoride electrode (Hydromet S.C., Gliwice, Poland). Fluoride concentrations were not measured for plant samples. All trace element concentrations were expressed in mg per kg dry mass (Table 1).

**Table 1:** Mean (± SE) concentrations and sample sizes for seven trace metals and fluoride in bone (mandible) and teeth (third permanent molar) of field and forest ecotypes of the European roe deer

Trace element	Bone				Teeth			
	Forest ecotype		Field ecotype		Forest ecotype		Field ecotype	
	Mean ± SE	*n*	Mean ± SE	*n*	Mean ± SE	*n*	Mean ± SE	*n*
Barium	204.1 ± 7.0	128	198.4 ± 7.6	91	196.9 ± 6.9	128	197.9 ± 7.8	92
Copper	4.73 ± 0.12	124	5.45 ± 0.22	91	4.56 ± 0.08	127	4.96 ± 0.17	88
Iron	18.15 ± 0.52	128	22.98 ± 0.77	90	17.15 ± 0.38	128	21.87 ± 0.55	91
Lead	0.32 ± 0.02	127	0.69 ± 0.05	91	0.29 ± 0.02	127	0.58 ± 0.05	91
Manganese	7.22 ± 0.58	121	6.07 ± 0.19	92	64.9 ± 5.6	125	65.0 ± 5.9	92
Strontium	86.5 ± 3.3	128	95.2 ± 2.1	92	88.6 ± 3.3	128	98.9 ± 2.8	92
Zinc	98.1 ± 1.6	128	94.1 ± 1.4	90	105.9 ± 1.8	128	109.1 ± 2.4	91
Fluoride	2.76 ± 0.30	134	4.95 ± 0.56	90	2.14 ± 0.23	133	3.42 ± 0.39	89

All concentrations are given in mg per kg dry mass

### Trace Element Distributions and Outlier Analysis

Since distributions of most trace element concentrations showed strong right-skewness (mean skewness 2.09 ± 0.58 [SE] and 2.47 ± 1.00 [SE] for bone/tooth and plant samples, respectively), we performed an outlier analysis on the dataset. We used conservative criteria (> 5 SD) for outlier detection. Outlier analyses were conducted separately for tooth and bone samples, while all plant species were analysed jointly. We identified between one and three outliers for the concentrations of barium, copper, and manganese in bone, while two outliers were identified for the copper concentration in tooth samples. Also, two outliers were identified for the concentration of barium in plants. No outliers were identified for any other measurement. All outliers were removed from the dataset. Measurements that retained strong (> 1) right-skewness after outlier removal were log-transformed to improve normality.

### Interspecific Variation in Plant Trace Elements

There were significant differences in trace element content of different plant species. Concentrations of all trace elements, except for lead, showed significant differences between plant species, as assessed with the analysis of variance (ANOVA). In most cases (copper, iron, lead, manganese, and strontium), trace element concentrations were lowest in pine (Table 2). Post-hoc Tukey HSD comparisons showed that iron, manganese, and strontium concentrations in pine were significantly lower than in all other plant species (all *P* < 0.05), whereas copper concentrations in pine were significantly lower compared with blackberry (*P* = 0.010). Zinc concentrations were lowest in blueberry (Table 2; Tukey comparisons with other plant species: all *P* < 0.05), whereas barium concentrations were lowest in birch (Table 2; nonsignificant differences in Tukey comparisons: all *P* > 0.05). Maximum trace element concentrations were found in blackberry (copper and iron), blueberry (manganese), birch (lead, strontium, and zinc), and pine (barium) (Table 2). Because of these differences, we included plant species as a fixed factor in all further analyses of trace element content in plants.

**Table 2:** Mean (± SE) concentrations for seven trace metals in four plant species collected from areas with low and high woodland cover

Woodland cover	Trace element	Silver birch	Scots pine	Blackberry	European blueberry
		Mean ± SE	Mean ± SE	Mean ± SE	Mean ± SE
Low	Barium	34.05 ± 4.37	123.5 ± 55.49	17.06 ± 3.51	54.65 ± 8.66
	Copper	6.68 ± 0.38	5.74 ± 0.30	8.71 ± 0.71	7.18 ± 0.71
	Iron	80.72 ± 6.43	48.31 ± 5.13	98.52 ± 8.81	75.67 ± 7.36
	Lead	0.54 ± 0.20	0.46 ± 0.29	0.48 ± 0.13	0.58 ± 0.18
	Manganese	1107.7 ± 186.1	210.8 ± 34.6	706.2 ± 143.5	1308.0 ± 295.9
	Strontium	14.60 ± 1.64	2.95 ± 0.45	9.31 ± 1.56	7.17 ± 1.75
	Zinc	200.9 ± 21.1	37.33 ± 3.18	44.84 ± 3.59	31.82 ± 3.38
High	Barium	35.46 ± 4.84	10.17 ± 3.37	93.21 ± 70.66	50.41 ± 6.82
	Copper	5.07 ± 0.42	4.55 ± 0.25	5.36 ± 1.00	5.40 ± 0.93
	Iron	75.96 ± 6.71	39.54 ± 4.09	95.03 ± 5.62	53.23 ± 3.22
	Lead	0.67 ± 0.41	0.16 ± 0.07	0.41 ± 0.24	0.37 ± 0.19
	Manganese	1437.1 ± 389.2	217.1 ± 58.1	1623.2 ± 692.2	2280.2 ± 554.2
	Strontium	12.09 ± 2.55	2.38 ± 0.29	14.27 ± 2.54	5.73 ± 1.34
	Zinc	216.3 ± 27.2	41.34 ± 2.44	58.11 ± 13.66	19.37 ± 1.93

All concentrations are given in mg per kg dry mass

### Statistical Analyses

We used general linear mixed models (GLMMs) to test for the ecotype variation in trace element content in bone and teeth of the roe deer. Ecotype, sample type (bone vs. teeth), and age were entered as fixed factors. To test whether ecotype-related differences in trace element content were similar for both sample types, we also entered an ecotype-sample type interaction in each model. As age-related bioaccumulation rate of trace elements can vary between bone and teeth of the roe deer (our previous research on roe deer provided support for positive correlations between trace element concentrations and age in teeth, but not in bone (Demesko et al. 2018)), we also included age-sample interaction to account for these differences. Because teeth and bone samples were collected from the same individuals, we included individual identity as a random factor to avoid pseudoreplication (Hurlbert 1984). The effect of year was included as the second random factor to control for interannual variation in the collected measurements. All GLMM models were fitted using the restricted maximum likelihood (REML) method. With this approach, denominator degrees of freedom are calculated using a Satterthwaite approximation, which can result in fractional degrees of freedom (Satterthwaite 1946). Significance of independent variables was assessed with Wald *χ*^2^ statistic.

Differences in trace element content of plants collected in the study plots with low (< 35%) and high (> 50%) woodland cover (consistent with field and forest deer ecotypes) were assessed with general linear models (GLMs). Plant species and the binary effect of woodland cover (low vs. high) were entered as fixed factors. To test whether the effect of woodland cover on trace element concentrations was similar for different plant species, we also included an interaction term between these two factors.

All GLMMs were run using lmer function as implemented in lme4 package (Bates et al. 2015) developed for R statistical environment (R Development Core Team 2013). We used *car* package (Fox and Weisberg 2011) to obtain Wald *χ*^2^ statistics and *p* values for all independent variables. GLMs were conducted in Statistica 10.0 (StatSoft, Tulsa, OK, USA). The results of full models were reported. All values are shown as mean ± SE.

## Results

**Fig. 1:**
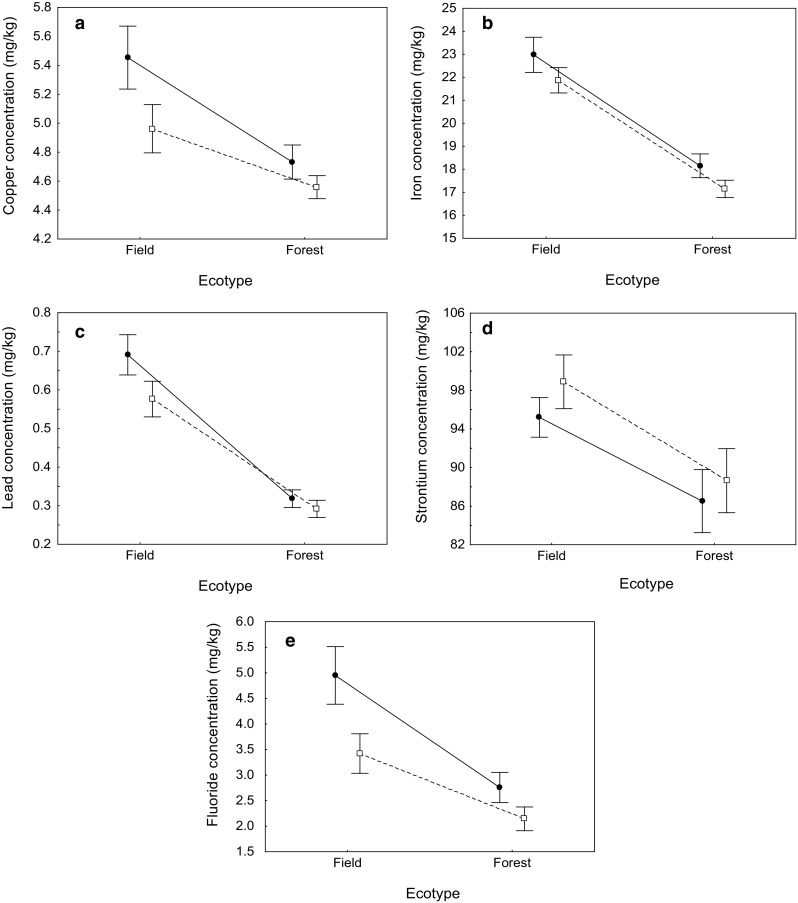
Concentrations of trace elements (**a** copper, **b** iron, **c** lead, **d** strontium, and **e** fluoride) in bone (solid line, filled circles) and teeth (dotted line, open squares) of the two ecotypes (field and forest) of the European roe deer. Mean ± SE are presented

After accounting for age-related variation in trace element content, we found that concentrations of four trace metals (copper, iron, lead, strontium) and fluoride in roe deer significantly varied with ecotype (Table 3). The effects of ecotype on the concentrations of these elements were similar for bone and tooth samples, as indicated by nonsignificant ecotype-sample type interactions (Table 1). In all these cases, trace element concentrations were significantly lower in the forest ecotype compared with the field ecotype, both in bone and teeth of roe deer (Table 1, Fig. 1). There was a significant ecotype-sample type interaction for zinc concentration (Table 3), but no significant effect of ecotype on zinc concentration was found in separate analyses of bone and tooth samples (all *P* > 0.05). Also, we failed to find any differences in the concentrations of barium and manganese between the forest and field ecotypes of roe deer.

**Table 3:** The results of general linear mixed models assessing the effect of ecotype and other factors (sample type and age) on the concentrations of seven trace metals and fluoride in bone and teeth of the European roe deer

Factor	Barium		Copper		Iron		Lead	
	*W*	*P*	*W*	*P*	*W*	*P*	*W*	*P*
Ecotype	0.08	0.77	**16.08**	**< 0.001**	**81.17**	**< 0.001**	**39.92**	**< 0.001**
Ecotype*sample type	1.37	0.24	1.91	0.17	0.01	0.93	3.35	0.067
Age	1.99	0.57	6.56	0.087	**44.58**	**< 0.001**	2.58	0.46
Age*sample type	**59.80**	**< 0.001**	1.15	0.77	4.46	0.22	7.45	0.059
Sample type	2.71	0.10	**16.41**	**< 0.001**	**4.10**	**0.043**	2.04	0.15

Factor	Manganese		Strontium		Zinc		Fluoride	
	*W*	*P*	*W*	*P*	*W*	*P*	*W*	*P*
Ecotype	0.49	0.48	**4.71**	**0.030**	0.37	0.54	**16.49**	**< 0.001**
Ecotype*sample type	1.52	0.22	0.30	0.58	**4.47**	**0.035**	1.26	0.26
Age	**9.97**	**0.019**	3.65	0.30	**58.66**	**< 0.001**	**21.18**	**< 0.001**
Age*sample type	**16.08**	**0.001**	**26.51**	**< 0.001**	**102.1**	**< 0.001**	**9.30**	**0.026**
Sample type	**729.1**	**< 0.001**	**6.47**	**0.011**	**67.23**	**< 0.001**	**14.28**	**< 0.001**

Individual identity was entered as a random factor in each model

Significant terms are marked in bold

**Fig. 2:**
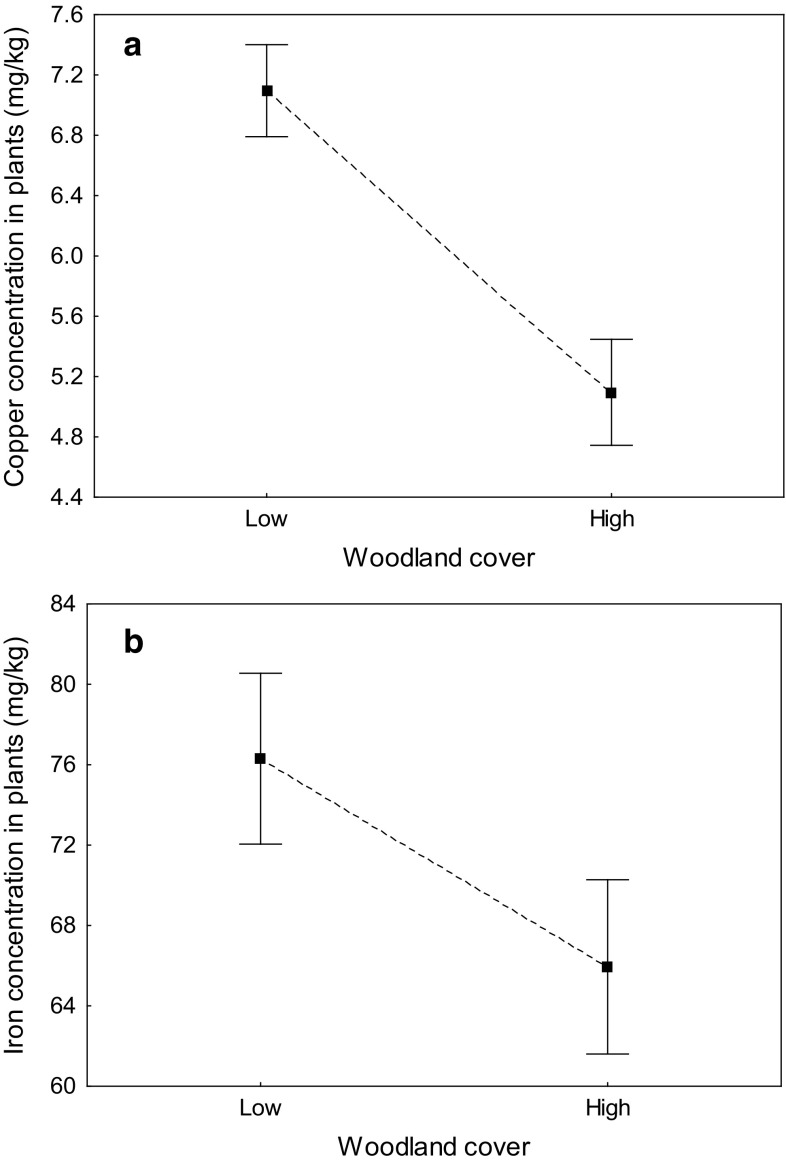
Concentrations of copper (**a**) and iron (**b**) in four wild plant species sampled in study plots with low (< 35%) and high (> 50%) woodland cover. Mean ± SE for all taxa combined are presented

Concentrations of two trace elements, copper and iron, in plants varied with woodland cover irrespectively of sampled plant species (Table 4). Specifically, plants collected in areas with lower woodland cover had higher concentrations of copper (7.10 ± 0.30 mg/kg vs. 5.10 ± 0.35 mg/kg) and iron (76.3 ± 4.2 mg/kg vs. 65.9 ± 4.3 mg/kg) (Fig. 2). Lead concentration in plants showed nearly significant variation with woodland cover (*P* = 0.061; Table 4), as plants from areas with lower woodland cover had a tendency for higher concentrations of lead (0.51 ± 0.10 mg/kg vs. 0.40 ± 0.13 µg/kg). Relationships between woodland cover and concentrations of two other trace elements, barium and zinc, varied between plant species, as indicated by significance of appropriate interaction terms (Table 4). Barium concentration in pine and zinc concentration in blueberry varied nearly significantly or significantly (respectively) with woodland cover (barium: *F*_1,21_ = 3.18, *P* = 0.089; zinc: *F*_1,19_ = 7.60, *P* = 0.013), and in both cases, element concentrations were higher in areas with lower woodland cover (barium: 123.5 ± 55.5 mg/kg vs. 10.2 ± 3.4 mg/kg; zinc: 31.8 ± 3.4 mg/kg vs. 19.4 ± 1.9 mg/kg). Barium and zinc concentration in other plant species did not vary with woodland cover (all *P* > 0.10). Manganese and strontium concentrations in plants did not differ between areas of low and high woodland cover (Table 4).

**Table 4:** The results of general linear models assessing the effect of woodland cover on the concentrations of seven trace metals in four wild plant species

Factor	Barium		Copper		Iron		Lead	
	*F*	*P*	*F*	*P*	*F*	*P*	*F*	*P*
Forest cover	0.33	0.57	**17.74**	**< 0.001**	**4.47**	**0.037**	3.59	0.061
Species	**6.30**	**< 0.001**	0.94	0.43	**29.37**	**< 0.001**	1.62	0.19
Forest cover*species	**3.47**	**0.020**	0.76	0.52	1.23	0.30	0.55	0.65

Factor	Manganese		Strontium		Zinc	
	*F*	*P*	*F*	*P*	*F*	*P*
Forest cover	0.93	0.34	0.01	0.94	0.24	0.62
Species	**12.88**	**< 0.001**	**28.62**	**< 0.001**	**1114.7**	**< 0.001**
Forest cover*species	0.55	0.65	1.65	0.18	**2.75**	**0.048**

Significant terms are marked in bold

## Discussion

The results of our study clearly indicate that field and forest ecotypes of the European roe deer showed significant differences in trace element content. Field roe deer had higher concentrations of four trace metals (copper, iron, lead, strontium) and fluoride in both bone and teeth compared with forest roe deer. Animal data were consistent with respective plant data, which indicated higher environmental contamination of areas inhabited by the field ecotype of roe deer.

It is widely accepted that animals living in heavily transformed habitats bear many costs, which are directly or indirectly associated with human activities (Gaillard et al. 1993; Benhaiem et al. 2008; Demesko et al. 2018). These costs may include direct human threat (e.g., hunting or poisoning with human waste; Kie 1999; Burbaitė and Csanyi 2009), pollution with light and noise that may cause elevated levels of physiological stress (De Vires 1995; Lima 1998; Pierce et al. 2004; Benhaiem et al. 2008), and exposure to novel predators, such as feral dogs and cats (May and Norton 1996; Apfelbach et al. 2005). The results of our study provide support for the hypothesis that intoxication with harmful substances of anthropogenic origin can be an important cost for wild animal populations from human-altered landscapes. So far, urbanization and human-related land alteration (e.g., intensive agricultural activities) has been associated with increasing intoxication level in a relatively wide spectrum of wildlife. For example, red foxes (*Vulpes vulpes*) and stone martens (*Martes foina*) from suburban area had higher content of copper and lead in soft tissues (muscle, liver, and kidney) compared with individuals originating from rural populations (Bilandžić et al. 2010). Analysis of trace element content in hair of three bat species indicated highest concentrations of lead and zinc in those species that collected food in human-dominated landscapes, including cities (Flache et al. 2015). Similarly, rook (*Corvus frugilegus*) eggshells from colonies located in large cities had significantly higher concentrations of chromium, nickel, and lead than those from villages (Orłowski et al. 2014). These results provided support for a huge variation in the habitat-dependent bioaccumulation of heavy metal in avian eggs, which occurred as a result of the clear pollution gradient from rural to urbanized areas (Orłowski et al. 2014). Lead and cadmium concentrations also were higher in blue tit (*Cyanistes caeruleus*) and great tit (*Parus major*) nestlings raised in urban parkland site than in the suburban semi-natural woodland site (Markowski et al. 2014). The levels of lead found in the tissues of urban house sparrows (*Passer domesticus*) were significantly higher than in an agricultural control group in Vermont, USA (Chandler et al. 2004) and Finland, where concentrations of several heavy metals were generally higher in urban than rural groups (Kekkonen et al. 2012). We are also aware of two previous studies on the European roe deer, which tested for associations between trace element content in deer tissues and habitat-related environmental contamination. First, roe deer from the major industrial sites in Poland had much higher (up to an order of magnitude) concentrations of lead and cadmium in tissues and stomach content than deer from the natural lake-forest ecosystems (Durkalec et al. 2015). The same study revealed similar patterns in trace element content of the wild boar (*Sus scrofa*) tissues (Durkalec et al. 2015). The second study provided evidence for higher concentrations of arsenic and chromium in teeth of roe deer that lived in a closer proximity to agricultural and industrial areas (Zaccaroni et al. 2008). We provided convincing evidence for high concentrations of several trace elements in the field ecotype of roe deer, which primarily feeds on crop fields and pastures. In contrast, trace element concentrations were significantly lower in the forest ecotype of deer, which prefers more natural ecosystems, such as various types of woodlands.

Five of eight measured trace elements (copper, iron, lead, strontium, and fluoride) had concentrations significantly higher in the field than forest ecotype of roe deer. Although the patterns were consistent between tooth and bone samples, we suggest that our results for fluoride should be treated with caution. The mean content of fluoride in the samples ranged from 2.14 ± 0.23 to 4.95 ± 0.56 mg/kg of dry matter, depending on the type of tissue and ecotype (Table 1). In contrast, fluoride concentrations previously reported for different wildlife ungulate and non-ungulate species coming from unpolluted European areas often achieved hundreds or thousands mg/kg of dry matter (Kierdorf et al. 2000, 2012; Jelenko and Pokorny 2010; Kalisinska and Palczewska-Komsa 2011; Vieira et al. 2005). On the other hand, our fluoride measurements yielded higher average values than reported by Sobota et al. (2011) for skull bone and antler samples from roe deer collected in West Pomerania, Poland (< 1 mg/kg d.m.). Also, many authors (Kierdorf and Kierdorf 2003, 2009; Dabkowska et al. 1995; Jelenko and Pokorny 2010) emphasized that environmental concentrations of fluoride have been significantly decreasing in the recent decades, which could possibly be responsible for low fluoride content in our samples.

In our study, large differences in trace element content between the two deer ecotype were consistent with differences in the general level of environmental contamination, suggesting that bone and teeth of the European roe deer can be used as a valid indicator of environmental pollution. We found that wild forest plant collected from areas with low woodland cover (characteristic for field ecotype of deer) showed significantly higher concentrations of copper and iron, and a nearly significantly higher concentration of lead. Also, concentrations of barium and zinc were higher in the areas with lower woodland cover but only in specific plant taxa (pine and blueberry, respectively). It is possible that differences in the environmental contamination between more and less wooded areas could be directly explained with various intensity of farming activities. It is widely known that chemicals (fertilizers and pesticides) commonly applied in agriculture can lead to the higher accumulation of elements, such as iron, manganese, copper, or zinc in the soil (Singh 1994; Kabata-Pendias 1995; Romic and Romic 2003; Micó et al. 2006; Martiniaková et al. 2011). Also, agriculture often is associated with bigger traffic, which can further increase air pollution (Bunce 1985; Sobota et al. 2011). Finally, trees act as biological filters, removing a lot of airborne particles and, at the same time, improving the quality of air in polluted areas (Nowak et al. 2006). Thus, in most cases a negative correlation between woodland cover and environmental contamination should be well expected (Beckett et al. 1998), resulting both from lower exposure to contaminants and their more efficient removal in the wooded areas.

We did not study differences in foraging by the two ecotypes. However, large differences in trace element content of wild plants sampled from predominantly agricultural and predominantly wooded study plots suggests that high concentrations of trace minerals in hard tissues of field roe deer may not be a direct consequence of variation in diet between the two ecotypes. Foraging strategy and diet composition differs considerably between the two ecotypes (Kałuziński 1982), whereby field roe deer predominantly forage on agricultural plants and herbs with a small share of bushes and trees, while forest roe deer prefer grasses, as well as shoots and bark of woody plants (Kałuziński 1974, 1982; Szmidt 1975). The two ecotypes also differ in many other aspects of ecology, which under certain scenarios could possibly affect their exposure of contamination. For example, forest deer has much smaller home range, which was estimated at only 3–8 ha in dense coniferous/deciduous woods, although it may be a few times larger in more fragmented landscape (Tufto et al. 1996). The two ecotypes also differ in the level of sociality (field deer form large winter groups of up to 100 individuals, while forest deer are more solitary; Zejda 1978; Bresinski 1982) and behaviour, e.g., antipredator strategies (Fruziński et al. 1983; Pielowski and Bresiński 1982; Aulak and Babińska-Werka 1990; Hewison et al. 2001). While it might be difficult to offer specific predictions about how these differences could affect trace mineral content in deer, we cannot exclude that factors, such as the size of home range and the level of sociality, for example, could determine movement propensity of animals across different habitats, which could possibly alter their exposure to environmental contamination.

## Conclusions

Our study provided correlational evidence for increased concentrations of trace elements in the field ecotype of the European roe deer, which primarily inhabits human-transformed agricultural landscape. Although the field ecotype of the European roe deer was first described in 1929 (Kałuziński 1974), its population size is thought to have considerably grown over the past decades, mainly due to the increasing fragmentation of forest habitats and greater availability of human-derived food. Future monitoring of European roe deer is warranted to explore ecotoxicological differences between field and forest roe deer.

## Electronic Supplementary Material

Below is the link to the electronic supplementary material.

Supplementary material 1 (XLSX 76 kb)
